# Clinical effects of breast milk enema on meconium evacuation in premature infants: study protocol for a randomized controlled trial

**DOI:** 10.1186/s13063-021-05261-1

**Published:** 2021-04-26

**Authors:** Liqiang Zheng, Li Gai, Jinyue Gao, Chaonan Kong, Yali Wang, Fangli Sun, Sitong Liu, Xinying Yu, Fan Yang, Hong Jiang

**Affiliations:** 1 Department of Pediatrics, Shengjing Hospital of China Medical University, Shenyang, 110004 People’s Republic of China; 2 Department of Library, Shengjing Hospital of China Medical University, Shenyang, 110004 People’s Republic of China; 3 Department of Clinical Epidemiology, Shengjing Hospital of China Medical University, Shenyang, 110004 People’s Republic of China; 4 Department of Epidemiology, School of Public Health, Shanghai Jiao Tong University, Shanghai, 200000 People’s Republic of China; 5 Department of Nursing, Shengjing Hospital of China Medical University, Shenyang, 110004 People’s Republic of China

**Keywords:** Breast milk enema, Extremely preterm infants and preterm infants, Complete meconium evacuation, Full enteral feeding, Randomized controlled trial

## Abstract

**Background:**

Delayed meconium evacuation is an important cause of intestinal dysfunction in preterm infants. There are many methods to induce defecation in preterm infants: however, the effects are controversial. Finding a new intervention method to promote meconium evacuation in premature infants is necessary. Therefore, in the proposed study, the effectiveness of breast milk enema on complete meconium evacuation and time to achieve full enteral feeding will be investigated in preterm infants.

**Methods/design:**

The study is a randomized, open-label, parallel-group, and single-center clinical trial. A total of 294 preterm infants will be recruited and stratified based on gestational age. Then, the infants will be assigned in a randomized block design to the intervention and control groups with a 1:1 ratio. Preterm infants in the control and intervention groups will receive saline enema and breast milk enema, respectively. The primary outcomes will be the time to achieve complete meconium evacuation from birth and time to achieve full enteral feeding from birth in preterm infants. The secondary outcomes will include hospitalization days, body weight at discharge, duration of total parenteral nutrition, cholestasis, and adverse events.

**Discussion:**

The results of this trial will determine whether breast milk enema shortens the time to complete meconium evacuation and the time to achieve full enteral feeding in extremely preterm and preterm infants. Furthermore, the study results may provide a new, safe, inexpensive, and easy-to-use intervention to effectively evacuate meconium in preterm infants.

**Trial registration:**

ISRCTN Registry ISRCTN17847514. Registered on September 14, 2019

## Background

According to estimates, 15 million premature infants are born in the world every year [1]. China is a country that has the second highest number of premature infants: 1.17 million premature births are reported in China every year, accounting for 10% of all newborns [2]. Premature infants have an immature intestinal motility mechanism and neurotransmitter system that delay meconium evacuation. The younger the gestational age of premature infants, the longer the time for the evacuation of meconium [3–6]. Because the meconium contains high levels of bilirubin, a delay in meconium evacuation increases the intestinal circulation of bilirubin, thereby aggravating neonatal jaundice and increasing the risk of bilirubin brain damage, kernicterus, serious sequelae, and even death [7]. Furthermore, delayed meconium evacuation is a recognized cause of intestinal dysfunction [8, 9], which can cause delayed total enteral nutrition, gastric retention, and feeding intolerance in preterm infants. A delay in total enteral nutrition is closely associated with an increase in the frequency of the incidence of necrotizing enterocolitis and mortality in premature infants [10–12]. In previous studies, promoting meconium evacuation was shown to improve feeding intolerance and promote weight gain in premature infants [13].

Currently, many methods exist to promote meconium evacuation [13–16], with the most popular being the use of enemas. Commonly used enemas include saline, glycerin solution, and glycerin suppositories. However, the effectiveness of glycerin solution and glycerol suppositories in promoting meconium evacuation was shown to be controversial in a meta-analysis [17]. Similarly, in a systematic review, saline was shown to not shorten the time to reach full enteral feeding in premature infants [18]. Therefore, finding a new enema to promote meconium evacuation in premature infants is necessary.

In a previous study, the osmotic pressure of breast milk was found to be suitable [19] as a benign stimulant of the digestive tract and with the added benefit of guaranteed safety. Saline is the most commonly used enema to promote defecation in clinical practice [20], and therefore, in the proposed study, a saline enema will be used in the control group. This study will explore whether breast milk enema can shorten the time of complete meconium evacuation and the time to reach full enteral feeding among premature and extremely premature infants.

### Objectives

In the proposed study, the effectiveness of breast milk enema on complete meconium evacuation and time to reach full enteral feeding will be investigated in premature infants.

## Methods

### Study design

**Fig. 1:**
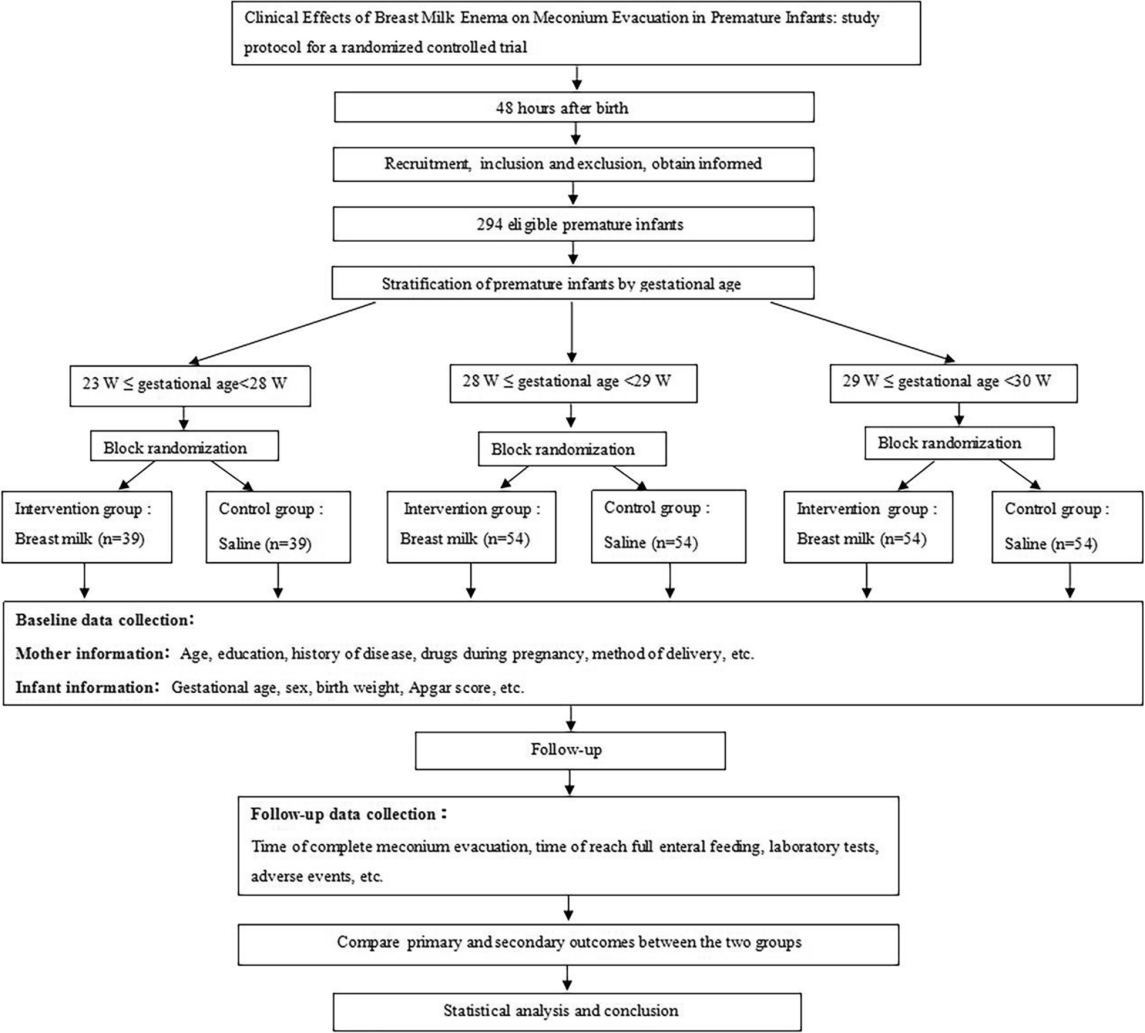
Study design of the project

The study will be a randomized, open-label, parallel-group, and single-center clinical trial. A total of 294 preterm infants will be recruited and stratified based on gestational age. Subsequently, the infants will be assigned, in a randomized block design, to the intervention and control groups with a 1:1 ratio. A flowchart of the trial procedure is shown in Fig. 1.

### Study setting

The trial will be performed in the Shengjing Hospital of China Medical University.

### Participants

To be eligible for inclusion in the trial, premature infants have to fulfill the following inclusion criteria: extremely premature infants (23 weeks ≤ gestational age < 28 weeks), premature infants (28 weeks ≤ gestational age < 30 weeks), mothers can provide breast milk 48 h postpartum, and the informed consent form is signed by the guardian. Premature infants meeting any of the exclusion criteria listed in Table 1 will be excluded.

**Table 1:** Participant inclusion and exclusion criteria

Criteria	Definition
Congenital malformations	Congenital malformations are structural or chromosomal malformations that have a major impact on health and development.
Congenital gastrointestinal anomalies	Congenital gastrointestinal anomalies are mainly diagnosed by ultrasonography or radiography. Congenital gastrointestinal anomalies include gastric volvulus, cecal volvulus, and heterotopic pancreas.
Anorectal deformities	Anorectal deformities are a wide spectrum of abnormalities of the anus and rectum, which include anal atresia and rectovesical fistula. Anorectal deformities will be diagnosed by magnetic resonance or sonography.
Diarrhea	Diarrhea is defined as the occurrence of 3 or more loose stools within 24 h.
Intussusception	Intussusception is characterized by telescoping of one part of the gastrointestinal tract into another part, forming an obstruction. Intussusception will be diagnosed by ultrasound.
NEC	The main symptoms of NEC are abdominal distension and hematochezia, which are characterized by intestinal mucosal necrosis. NEC is mainly diagnosed by abdominal X-ray and ultrasound examination.
PDA	PDA will be confirmed by echocardiography.
Sepsis	Sepsis includes early onset and late onset. Early-onset sepsis is caused by pathogens transmitted from mothers, and late-onset sepsis is caused by nosocomial infections. Sepsis is defined as the overgrowth of bacteria in blood cultures in the presence of clinical deterioration.
Neutropenia	Absolute neutrophil count < 0.5 × 10^9^/L.
Coagulopathy	International standardization ratio > 1.4, partial thromboplastin time > 39 s, fibrinogen < 1.00 g/L, and platelet count < 100 × 10^9^/L.

*NEC* necrotizing enterocolitis, *PDA* patent ductus arteriosus

### Recruitment

Leaflets with the trial information will be displayed on site in the waiting room of the neonatology department. A research assistant will ask each participant’s guardian if they would be interested in having their babies participate and will be given sufficient time to make their decision. The guardian can contact the research assistant using the provided telephone number and undergo screening to enter the trial.

### Informed consent

If the guardian is willing and the infant is eligible to participate in the study, the participant’s guardian will be required to sign a written informed consent. Informed consent procedures will ensure that the participant’s guardian understands participation is voluntary and that participants can withdraw from the study at any time. The informed consent can be obtained from the corresponding author.

### Additional consent provisions for collection and use of participant data and biological specimens

The main trial consent asks mothers of participants if they would be willing to allow the use of their baby’s data if they choose to withdraw from the trial. This trial does not involve collecting biological specimens for storage.

### Randomization

Participants will be allocated to the intervention and control groups with stratified block randomization using a computer-generated randomization list by an independent statistician not otherwise involved in the trial. The participants will be stratified based on gestational age. Then, the participants will be assigned in a randomized block design to the intervention and control groups in a 1:1 ratio. The block size will be 4 and 6 to ensure equal numbers of participants in the intervention and control groups. At the beginning of the trial, a random sequence will generate a set of study numbers. Each study number will be paired with a method of intervention and sealed in an opaque envelope. The envelopes will be grouped based on gestational age. Recruited participants will be allocated to the intervention or control groups by selecting an envelope.

### Blinding

The design is open-label with only outcome assessors and data analysts being blinded.

### Intervention

**Fig. 2:**
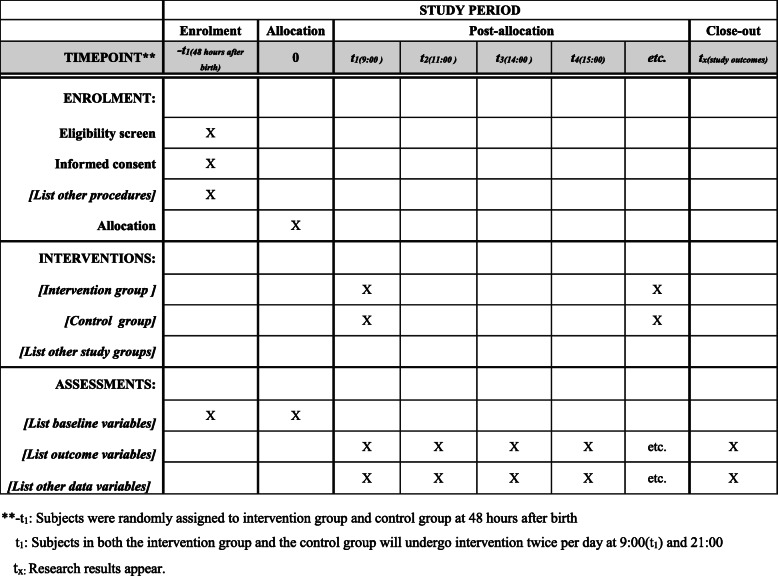
The schedule of enrollment, interventions, and assessments

Preterm infants in the control and intervention groups will receive a saline and breast milk enema, respectively. Nurses will be trained to ensure the standardized and safe administration of enemas. The timeline of the trial is shown in Fig. 2. The specific procedures are described as follows:

1. Meconium evacuation procedure: preterm infants in intervention and control groups will undergo meconium evacuation twice per day at 9:00 and 21:00. The intervention will continue until the meconium is completely evacuated.
2. Preparation of materials: silicone tube (4–6 cm, model 3.33 mm [F10]), 5 mL syringe, thermostat (temperature setting 37 °C), sesame oil, sterile gloves.
3. Implementation: the number of enemas required will be calculated (5 mL/kg) and preheated to 37 °C in a thermostatically controlled water bath. The preheated enemas will be extracted with a syringe. One end of the silicone tube will be connected to the syringe, and the other end will be lubricated with sesame oil before inserting it 2–3 cm into the rectum. The enema will be slowly injected into the rectum and will be retained there for 3 min; the silicone tube will then slowly be pulled out.

If a premature infant does not pass stools during the 24 h following the complete evacuation of meconium, an enema will be administered. A saline enema will be used once a day in both intervention and control groups using the same method as described above.

The criteria for the termination of intervention will include complete meconium evacuation, the development of any of the serious adverse events listed in the “Study outcomes” section, or the withdrawal of a guardian’s consent.

To improve adherence and promote participant retention, nurses and doctors will be trained in study-specific procedures prior to participation in the study. In addition, guardians will be updated weekly on the status of their infants by a nurse who is otherwise not involved in the study. The Trial Steering Committee (TSC) will also dynamically monitor the rate of follow-up loss.

### Feeding procedures

The same feeding and parenteral nutrition procedures will be used for premature infants in the control and intervention groups. The total daily calories of enteral and parenteral nutrition will be 110–135 kcal/kg. Premature infants will be fed starting at 24 mL/kg birth weight per day. Furthermore, feedings will be advanced every 24 h by 20 mL/kg birth weight per day. When the amount of enteral feeding reaches 120 mL/kg per day, parenteral nutrition will be stopped. We defined full enteral feeding as tolerance of enteral feeding volumes of 180 mL/kg/day within 24 h and a weight gain greater than 20–25 g/d within 24 h. Human milk feeding will be encouraged. If the amount of breast milk is insufficient to meet the nutritional needs of premature infants, mixed feeding (artificial and breast milk) will be adopted. We will use disposable bottles for feeding to calculate the volume of feeding and use nasogastric feeding for premature babies with poor sucking ability. Feeding will be stopped if feeding intolerance occurs, such as when a gastric residual volume occurs that is more than half of the feeding volume, an abdominal circumference that is increased by 2 cm more than before the previous feeding, or with the occurrence of an intestinal dynamic anomaly.

### Study outcomes

#### Primary outcomes

The primary outcomes will be the time to achieve complete meconium evacuation from birth and the time to achieve full enteral feeding from birth in preterm infants.

#### Secondary outcomes

Secondary outcomes will include hospitalization days, weight at discharge, duration of total parenteral nutrition (TPN), cholestasis during hospitalization, and adverse events (death or infection during hospital stay, retinopathy of prematurity (ROP, any stage), intraventricular hemorrhage (IVH, grade 2 and above), NEC, chronic lung disease (CLD)_,_ bronchopulmonary dysplasia (BPD), sepsis, malabsorption, diarrhea, fecal occult blood positivity, rectal bleeding, anorectal trauma, and colonic perforation).

### Data collection

The research data will be collected by trained researchers using standardized questionnaires and measurements to ensure accuracy. At baseline, the following information regarding premature infants and their mothers will be collected: gestational age, birth weight, demographic information, history of disease, and medications used during pregnancy. The following major aspects of outcome information will be collected during the follow-up: meconium evacuation, feeding, laboratory tests, and adverse events.

### Data management and quality control

All extremely preterm infants and preterm infants will be given an individual study ID based on the same rules. The data will be securely managed throughout the trial process and reviewed by quality control experts. Reasons must be given when data are modified. Data will be double-entered using the Epidata database. The locked database will be used for analysis.

### Sample size and statistical power estimation

The sample size estimate is based on our pilot study; the time of complete meconium evacuation was 1.4 days, 1.2 days, and 1.2 days earlier in the breast milk enema group than in the saline enema group at 23 weeks ≤ gestational age < 28 weeks, 28 weeks ≤ gestational age < 29 weeks, and 29 weeks ≤ gestational age < 30 weeks, respectively. Given that *α* = 0.05, *β* = 0.2, standard deviation (SD) = 2, sampling ratio = 1, and taking a 20% drop rate into account, the required total sample size is 78, 108, and 108 premature infants in each gestational age, respectively. A website was used to calculate the sample size (http://powerandsamplesize.com/Calculators/).

### Missing data

The relevant researcher will contact the premature infant’s guardian by telephone or check electronic medical records to supplement missing baseline and follow-up data. If no reply is received from the guardian or missing data cannot be supplied, the missing data will be processed using different methods based on the mechanism of the missing data. For example, the mean value imputation and last observation carried forward will be used to supplement the data. Furthermore, we will conduct a sensitivity analysis to evaluate the robustness of the trial results: when we analyze the primary outcomes, we will conduct a comparative analysis of complete (ignoring the missing data) and incomplete (no missing data) cases.

### Statistical analysis

We will conduct intention-to-treat and per-protocol analyses. Baseline descriptive data will be compared between the intervention and control groups using a *t* test or chi-square test. The primary outcomes will be analyzed using an independent sample *t* test or Mann-Whitney test. Meanwhile, a multiple linear regression model will be used to analyze the factors affecting the primary outcomes, including gender, birth weight, and history of a mother’s disease. In addition, we will perform a subgroup analysis based on gestational age to compare the main outcomes of the two groups.

A two-sided *P* < 0.05 will be considered statistically significant. All analysis will be performed in the SAS version 9.2 (SAS Institute Inc., Cary, NC, USA) and SPSS version 22.0 (IBM Inc., Chicago, IL, USA) software.

### Interim analyses

Interim analyses are not planned.

### Oversight and monitoring

#### Composition of the coordinating center and Trial Steering Committee and composition of the Data Monitoring Committee, its role, and reporting structure

This trial has been supervised by the TSC, which consists of experts in epidemiology, pediatrics, and infectious diseases. The main responsibilities of the committee include the supervision of the study design, implementation, data analysis, and trial reports. The TSC will meet every 3 months. The Data Coordination Center (DCC) will mainly be responsible for the quality control of data and the implementation of randomization. The Data Security Monitoring Committee (DSMC) will monitor and record any security issues that occur with regard to participants during the trial and propose solutions. The DCC and DSMC will meet once a month. Furthermore, the DCC and DSMC will report to the TSC. Stakeholder and public involvement groups will not be permitted.

### Adverse event reporting and harms

All adverse events observed by the investigators will be recorded and reported to the DSMC, highlighting any seriousness as required. Furthermore, any causality between intervention and adverse events will be recorded.

### Frequency and plans for auditing trial conduct

The Trial Management Team will meet every 3 months to review the implementation of the study. The independent monitor will make an on-site visit once a month and check the quality of the data, including the inclusion and exclusion criteria, informed consent, original data, and the absence of data.

### Plans for communicating important protocol amendments to relevant parties

A substantial amendment is defined as an amendment to the protocol that is likely to have a significant effect on the safety of participants. Notification of all substantial amendments will be provided to the participants’ guardians and ethics committees. Non-substantial amendments and any deviations from the protocol will be fully recorded using a breach report form. In addition, online trial registries will be updated accordingly.

### Dissemination plans

The results of the study will be completely reported in international peer-reviewed journals. Both positive and negative results will be disclosed. We hope that our findings provide a potential clinical significance.

### Biological specimens

Biological specimens will not be collected in this trial.

### Confidentiality

During the course of the study, data collected will be kept strictly confidential and only accessed by members of the trial team. Data will be stored in a secure database. In publications, no identifying details of participants will be reported. We will determine whether anonymized trial data can be used for meta-analyses based on discussion by the ethics committee.

## Discussion

Due to the large number of premature infants being born worldwide, countries are facing an enormous medical burden. Feeding is a significant challenge for premature infants, and establishing full enteral feeding is an important goal in their care. Earlier full enteral feeding is associated with fewer failures of postpartum growth. Unfortunately, any delay in meconium excretion is related to a delay in transition to full enteral feeding. Compared with term infants, many premature infants pass the first meconium only after considerable delay. Times for the evacuation of the first and last meconium are critical for gastrointestinal function and feeding tolerance. If meconium evacuation can be expedited through the use of an effective enema, this may lead to a faster transition to full enteral feeding and a decreased reliance on intravenous nutrition. However, the available evidence is inconclusive. In contrast to other enemas, the amount of magnesium salts in breast milk is high and relatively stable, which can promote gastrointestinal peristalsis and help meconium evacuation [21, 22]. In addition, breast milk can reduce the risk of enterovirus infection and balance the intestinal flora of premature infants [23, 24]. Therefore, the trial will collect all participants’ data to determine whether this trial has potential clinical significance and can provide reliable evidence for clinical intervention. The results may yield a new, safe, inexpensive, and easy-to-use intervention to effectively evacuate meconium in preterm infants.

To the best of our knowledge, the major strength of this randomized controlled trial is to evaluate, for the first time, the effectiveness of a breast milk enema in promoting complete meconium evacuation and reach full enteral feeding among preterm and extremely preterm infants. Consequently, the results will provide critical information regarding a new enema to effectively evacuate meconium in preterm infants.

The study protocol has several limitations. First, some infants achieving complete meconium evacuation may withdraw before observing the time to reach full enteral feeding. We will further analyze and design a more comprehensive investigation based on this study. Second, this trial is a single-center clinical trial. Therefore, the representativeness of the participants is limited to some extent. Finally, some mothers may be unable to produce enough breast milk in early postpartum, which may slow down trial progress and prolong the time to recruit the estimated sample size.

### Trial status

Protocol version 1.0 and February 20, 2019. Recruitment to this study began in September 2019 and is expected to end in September 2022.

## Data Availability

The datasets analyzed during the current study are available from the corresponding author on reasonable request.

## Abbreviations

BPD: Bronchopulmonary dysplasia
CLD: Chronic lung disease
DCC: Data Coordination Center
DSMC: Data Security Monitoring Committee
IVH: Intraventricular hemorrhage
ITT: Intention-to-treat
NEC: Necrotizing enterocolitis
PDA: Patent ductus arteriosus
PP: Per-protocol
ROP: Retinopathy of prematurity
TPN: Total parenteral nutrition
TSC: Trial Steering Committee
